# Management Outcome of Adult Thyroglossal Duct Cyst in a General Surgery Tertiary Practice in Sub-Saharan Africa, Nigeria

**DOI:** 10.7759/cureus.31833

**Published:** 2022-11-23

**Authors:** Adefemi Afolabi, Adegbolahan Fakoya, Oluwasanmi Ajagbe, Adegoke Ishola

**Affiliations:** 1 Endocrine Surgery, University of Ibadan, Ibadan, NGA; 2 Endocrine Surgery, University College Hospital, Ibadan, NGA; 3 Surgery/Oncology, University College Hospital, Ibadan, NGA; 4 Surgery, Nigerian Army Medical Corps, Lagos, NGA; 5 Surgery, University College Hospital, Ibadan, NGA

**Keywords:** nigeria, ibadan, adult, tdc, sistrunk operation

## Abstract

Thyroglossal duct cyst (TDC) is a rare condition in adults. This is a report of its presentation and standard treatment with good outcome in adults in a Sub-Saharan tertiary hospital.

It is a retrospective review of five adult patients who were managed over a five-year period culled from archived records and casenotes. The three males and two females have a median age of 35 years and a range of 15-74 years. Each of them presented with anterior neck masses while one, in addition, had a painful swelling with a discharging sinus. The median duration of the symptoms was 4.5 years. Four of the cysts were in the infrahyoid location and all the patients had a Sistrunk operation. The median duration of follow-up was six months, without complications. Surgeons need to have a high level of suspicion for this condition in adults.

## Introduction

The thyroglossal duct runs from the foramen caecum at the junction of the anterior two-thirds and the posterior third of the tongue to the adult anatomical location of the thyroid gland [1]. The occurrence of thyroglossal duct cyst (TDC) occurs because of persistence of the embryonic thyroglossal duct [2].

TDCs often occur in the paediatric age group with fewer cases reported in adulthood [3]. The majority of the published literature focuses on its management in children and most are often benign, but 1% may be malignant [3,4].

Sistrunk procedure is recognized as the standard operation for the management of TDCs globally. The procedure involves excision of the cyst, the central portion of the hyoid bone, and the tract up to the foramen cecum. This procedure follows the path of the embryologic thyroglossal duct relative to the hyoid bone which is the fulcrum for its existence [5]. The Sistrunk procedure is the same in both children and adults and offers good outcome, more so in documented evidence in children and limited outcome data in adults [6]. Due to the limited report on outcome of surgical management in adult cases in comparison to children, we present the outcome of surgical care of diagnosed cases of thyroglossal cyst undertaken using the Sistrunk procedure.

## Case presentation

A total of five patients were managed over a five-year period, and we describe the experience with the management of these adults with TDC in a Nigerian Reference Hospital. This is a retrospective review of the clinical records of patients diagnosed and managed with histopathological evidence of TDC over a five-year period between June 2017 and February 2022 in the General Surgery Division of the Department of Surgery in the University College Hospital, Ibadan, Nigeria. The facility is a reference center for other institutions at different levels in Nigeria. The patients' case notes, and theatre records were reviewed. The clinical characteristics including the symptoms, length of symptoms, other signs; preoperative work up both biochemical and radiological; cadre of surgeon, operative technique, operative findings, surgical outcome, and pathology reports were reviewed. Data were analyzed using Statistical Package for Social Sciences computer software (SPSS) version 22 (IBM Corp., Armonk, NY, USA) for descriptive analysis. The results are presented in tables and figures.

There was a total of five patients managed for TDC over the stated period. There were three males and two females with an age range of 15-72 years. Their median age was 35 years.

**Figure 1:**
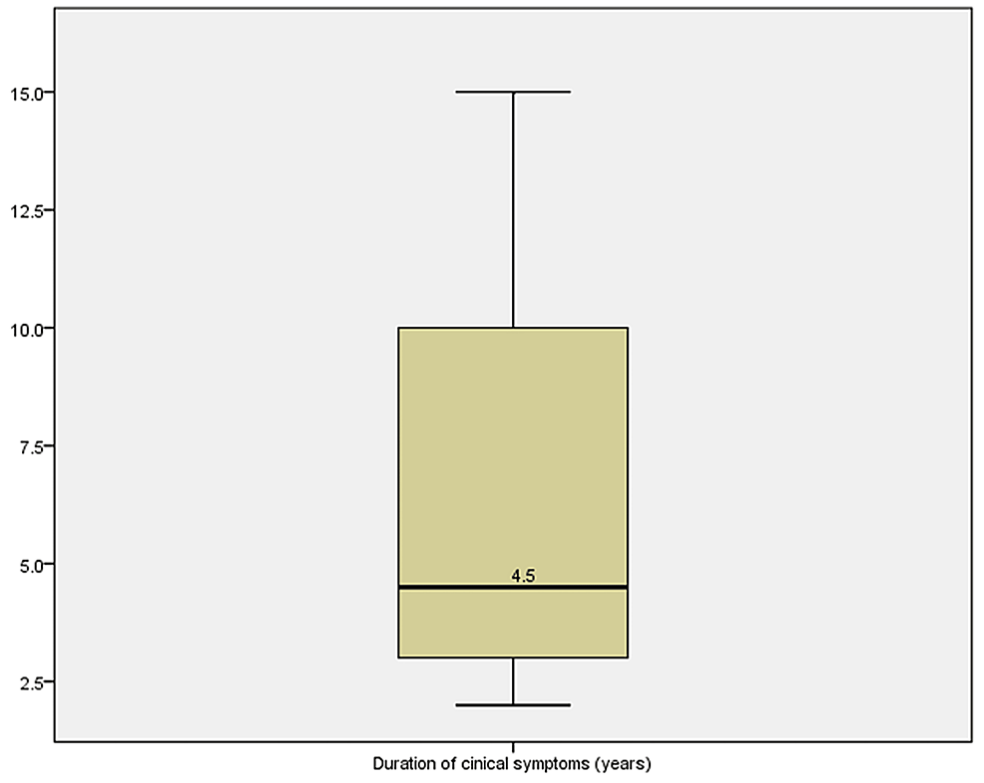
Box plot showing duration of symptoms

**Figure 2:**
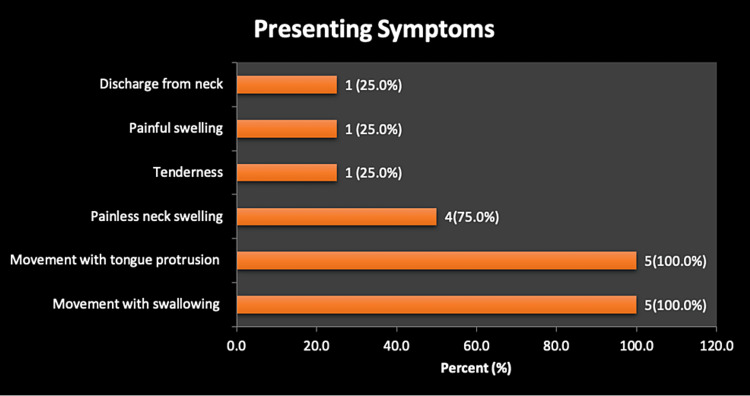
Presenting complaints

All the patients presented with anterior neck swelling with a median duration of 4.5 years (range 2-15 years) (Figure 1). One patient presented with discharge from the anterior neck and pain (Figure 2). The thyroid function tests done on all the patients were normal.

**Figure 3:**
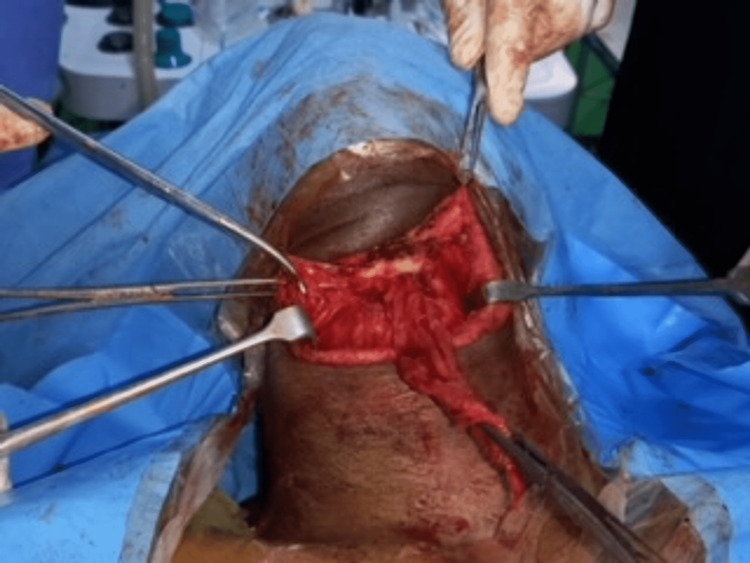
Mid portion of the hyoid bone at surgery

**Figure 4:**
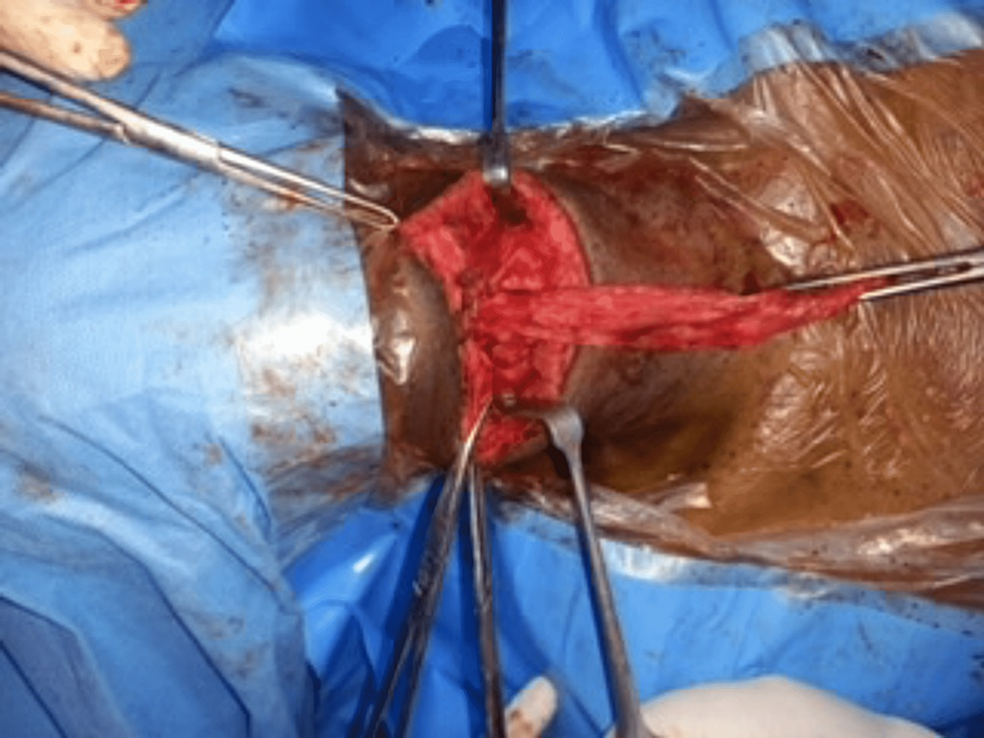
Ruptured thyroglossal cyst in a 72-year-old man at surgery

The oldest patient (a 72-year-old male) presented with a 15-year history of painless anterior neck swelling and co-morbidities (hypertension, diabetes, Parkinson disease, stroke survivor, previous craniotomy), at presentation, elderly man, with an obvious anterior neck swelling that moved with deglutition and tongue protrusion. a clinical diagnosis of thyroglossal duct cyst was made. Intra-operative pictures below shows the ruptured cyst at surgery (Figures 3, 4).

All the patients had preoperative neck ultrasound with major finding of hypoechoic lesion in the midline of the neck with normal thyroid gland. Preoperative thyroid function tests were normal in all the patients. Four of the patients had preoperative fine needle aspiration cytology the reports of which were benign.

**Figure 5:**
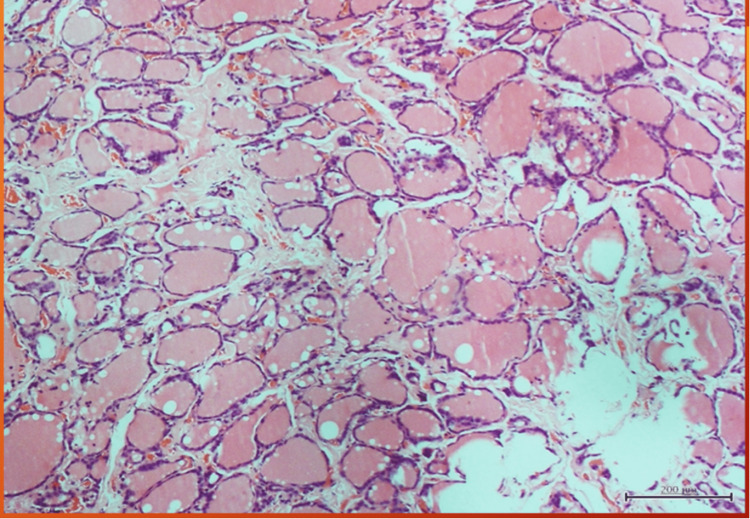
Shows (X40) H&E section- thyroid follicles lined by single layer of columnar epithelial cells filled with colloid, no malignant cells seen in the 72-year-old patient.

**Figure 6:**
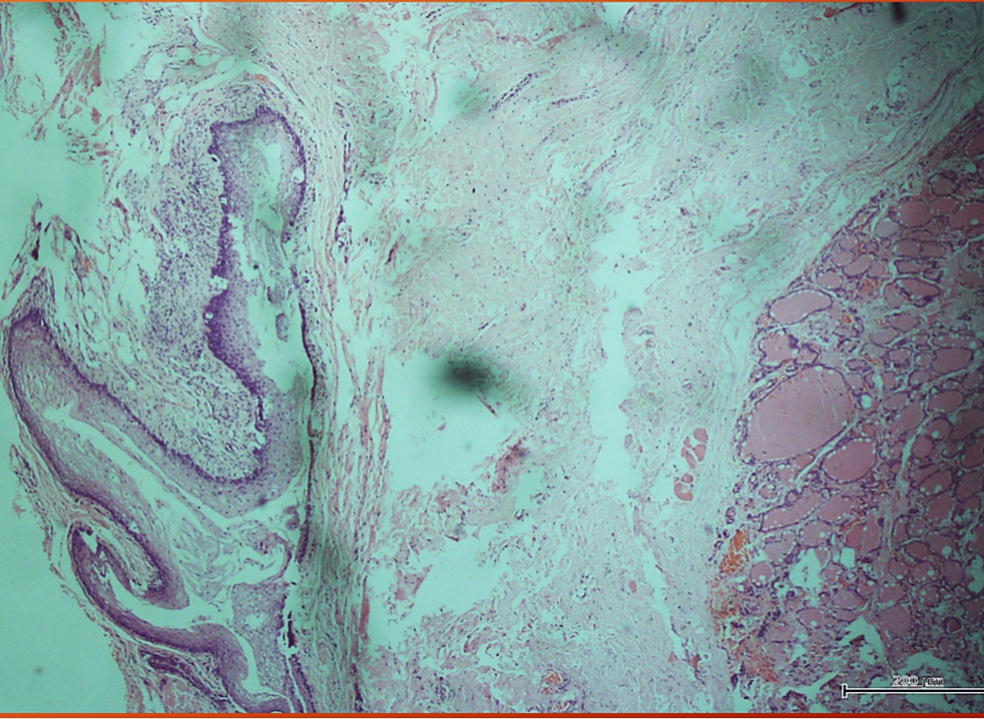
Shows fragments of tissue lined in areas by stratified squamous epithelium (left) and the fibrocollagenous wall shows various sizes of thyroid follicles filled with colloid (right) in the 72-year-old male patient

Four of the cysts were in the infrahyoid position while one was in the suprahyoid position and all were located in the midline. Sistrunk operation was performed by a consultant surgeon in each of the patients (Table 1). The procedure entailed excision of the cyst, and excision of the mid portion of the hyoid bone. The duration of hospital stay was one to three days and there were no recurrences at the sixth month of follow-up. The histopathology report confirmed TDC in all patients and none was malignant. Figure 5 and Figure 6 show photomicrographs of the specimen of one of the patients with TDC and no malignant cells were seen.

**Table 1:** Characteristics and surgical intervention of the cysts

Variables	Frequency (%)
Nature of cyst	
Benign	5 (100.0)
Malignant	0 (0.0)
Diagnosis	
Thyroglossal duct	5 (100.0)
Type of procedure	
Sistrunk procedure	5 (100.0)

## Discussion

TDC is rare in the adult population with a subsequent dearth of its report in the literature. Each of the few reports from Africa comprised a few cases respectively. The five patients in this report exemplify its rarity in adults, despite the referral status of this centre.

The age range of 15-74 years is consistent with that of most reviews from Africa [7]. While the sex distribution (three males and two females) in this report is consistent with the ratio of 1:1 in other publications in the literature [8], the actual gender distribution may be unknown due to the limited number of cases and the variation in gender distribution [7].

TDC has a bimodal age distribution with peaks at the first and fifth decades of life. This review has a patient in the seventh decade suggesting that it may also be common in the adult population [6]. The patients presented late from the time swelling was noticed with a median duration of presentation of 4.5 years. This may in part be due to the slow progression in the size of the cyst or earlier trial of alternative option of care apart from surgery as it is common in our clime due to fear of surgery.

The most reported location of the cyst is the infrahyoid position [3,9], with majority of the patients in this series presenting with an infrahyoid mass like the others in the literature. Presentation is usually as a painless swelling in the anterior neck [9], occasionally, pain and a discharging sinus may be the presentation [10] as it was in one of our patients who had an incision and drainage at a primary care level prior to the referral to this centre.

Four of the patients had preoperative fine needle aspiration cytology (FNAC). This might have necessitated total thyroidectomy in addition to the Sistrunk procedure and possible adjuvant therapy if malignancy had been diagnosed [10,11]. Papillary thyroid carcinoma could be found in 1% of TDC [10]. This makes it imperative to include FNAC in the preoperative assessment. Largely, the diagnosis of TDC is clinical [12], while laboratory and radiological investigations help with operative decisions.

The preoperative radiological investigation done in all our patients was neck ultrasound scan as it is the most available method for the assessment of neck anomalies [13], considering its availability and cost-effectiveness in our practice. The thyroid gland in all the patients were normal in consistency and location and this is important as diagnostic dilemma between TDC and ectopic thyroid tissue has been documented, culminating in severe complications of management [10]. Other radiological investigations such as computerized tomography and magnetic resonance imaging may be indicated in cases with atypical presentations [14,15].

Most ectopic thyroid tissue within TDC has a normally located and developed thyroid gland [16], which is euthyroid, while about a third may be hypothyroid preoperatively [17]. This justifies preoperative routine thyroid ultrasound and thyroid function tests. All the patients in this study had Sistrunk operation which is the documented standard technique for TDC [7]. While other surgical methods exist; they are all associated with high recurrence rate with simple cystectomy reported as high as 100% [6].

Newer approaches include robot-assisted, endoscopic procedure through transoral, retroauricular routes in the adult [18]. The advantage of these procedures besides cosmesis, over the traditional Sistrunk operation, remains in contention. Even then the procedures are not performed at present in our centre considering the large size of the cyst at presentation. The most documented complication is recurrence in up to 3-5% following a standard Sistrunk operation [8,19]. The five patients were followed up for more than six months with no recurrence. Most recurrence occurs within the first six months of surgery [10]. None of the patients had injury to the recurrent laryngeal nerve.

Other rare complications include damage to the hypoglossal nerve due to its proximity to the hyoid bone. The nerve is avoided with careful dissection and sparing of the superior horn of the hyoid bone [6,7]. Post-operative infections were not recorded in any of the patients although its incidence has been reported to be as high as 40% [6]. Most of our patients were discharged home between post-operative days one and two except the oldest patient who had presented with comorbidities.

## Conclusions

Surgeons need to have a high level of suspicion for this condition in adults. The prompt referral to a specialist is imperative for standard operation rather than incision and drainage which carries high recurrence rate. The outcome with the Sistrunk operation is good. A multicenter study within the subregion would further enhance the scope of studying this rare entity in adults.
